# Sequence-specific RNA Photocleavage by Single-stranded DNA in Presence of Riboflavin

**DOI:** 10.1038/srep15039

**Published:** 2015-10-13

**Authors:** Yongyun Zhao, Gangyi Chen, Yi Yuan, Na Li, Juan Dong, Xin Huang, Xin Cui, Zhuo Tang

**Affiliations:** 1 Natural Products Research Center, Chengdu Institute of Biology, Chinese Academy of Sciences. Chengdu 610041, P.R. China

## Abstract

Constant efforts have been made to develop new method to realize sequence-specific RNA degradation, which could cause inhibition of the expression of targeted gene. Herein, by using an unmodified short DNA oligonucleotide for sequence recognition and endogenic small molecue, vitamin B2 (riboflavin) as photosensitizer, we report a simple strategy to realize the sequence-specific photocleavage of targeted RNA. The DNA strand is complimentary to the target sequence to form DNA/RNA duplex containing a G•U wobble in the middle. The cleavage reaction goes through oxidative elimination mechanism at the nucleoside downstream of U of the G•U wobble in duplex to obtain unnatural RNA terminal, and the whole process is under tight control by using light as switch, which means the cleavage could be carried out according to specific spatial and temporal requirements. The biocompatibility of this method makes the DNA strand in combination with riboflavin a promising molecular tool for RNA manipulation.

Site-selective RNA cleavage to inhibit expression of special genes is consistently pursued by scientist for the promising application in biotechnology and therapy as well as biology. Previous research focused on gene suppression strategies through RNA degradation involving antisense oligonucleotides and artificial ribonucleases^1^,^2^,^3^,^4^,^5^. More efforts are still need to put into these methodologies to improve the efficiency before they could be broadly adapted as genomic or therapeutic tools. Since the first description of RNA interference (RNAi), there has been rapid progress towards its application as a therapeutic modality^6^. The mechanism of the RNAi is that the RNAi (the length of RNA must be longer than 21 bases), generated from double-stranded RNA, leads to specific degradation of complementary mRNA sequences^7^, triggers silencing of homologous gene expression^8^. Therefore, RNAi requires cell’s own machinery to fulfill its job. Ribozymes found in nature^9^,^10^ and deoxyribozyme obtained from *in vitro* selection^11^,^12^ could high-efficiently cleave the targeted RNA sequence-specifically through formation the right tertiary structure, which usually comprises of a catalytic domain which are flanked by two substrate-recognition domains.

Dilara Crate and Charles Wilson reported a site-specific inactivation of RNA transcripts through Laser-induced hydrolysis of the malachite green (MG)-binding RNA, which extend the RNA cleavage into a new scenario, namely under tight spatial and temporal control^13^. The application of this method is facing several challenges: (1) How to Introduction of the MG-binding motif into the 5′ untranslated region (UTR) of target RNA. (2) Toxicological effects of malachite green when applied in therapy. Herein, by using an unmodified short DNA oligonucleotide as binder and endogenic small molecue, vitamin B2 (riboflavin) as photosensitizer, we report a sample strategy to realize the sequence-specific photocleavage of targeted RNA.

Riboflavin, as the most efficient natural photoshensitizer, has been shown to induce photooxidative DNA damage via the generation of an 8-hydroxydeoxyguanosine intermediate^14^,^15^,^16^,^17^. The flavin-oligonucleotide conjugates have been successfully applied in the photocleavage of double stranded DNA target. In 1997, Famulok group reported that riboflavin could cleave RNA molecules with overwhelming specificity at G•U wobble via a photo-induced mechanism when they study an RNA aptamer for isoalloxazine derivatives which was obtained from *in vitro* selection^18^,^19^,^20^. As shown in Fig. 1A, they found the riboflavin could specifically recognize the G•U wobble in RNA duplex and induce the oxidative cleavage reaction through photosensitization. The Cleavage occurred independently of the residue located at the 3′ of the U (red, Fig. 1A), and the nucleoside downstream of the uracil (U) of the G•U base pairing was verified to be removed through oxidative mechanism. Their work inspires us to ask questions: How about the RNA/DNA duplex containing G•U wobble pair? Can any RNA sequence be photocleaved by DNA strand in presence of riboflavin?

As shown in Fig. 1B, a DNA strand was designed to complimentary to RNA target sequence except for forming a G•U wobble pair at the 17 nt position. The photocleavage of RNA target was verified by the isotope-labeled experiments (Fig. 1C). The 5′ ^32^P-labeled RNA target with 26 nt in length was incubated with the DNA sequence in presence 200 μM of riboflavin at 25 °C. After 4 h of irradiation with 45 W household light, a clear cleaving fragment with 17 nt in length was observed based on the PAGE gel analysis. Meanwhile, no cleavage could be observed when the DNA strand, riboflavin or light irradiation was absent in the cleaving reaction (Lane 1–3, Fig. 1C). To verify the importance of G•U wobble pair for the cleavage, the full complementary DNA sequence of RNA target was applied into the same reaction (Fig. 1B). As illustrated in the lane 4 of PAGE analysis in Fig. 1C, 5′ ^32^P-labeled RNA target kept intact after 4 h irradiation under the same reaction condition in presence of full complementary DNA strand, which indicates riboflavin could recognize the G•U wobble in RNA/DNA duplex to induce the site-selective photocleavage of RNA target.

The **c**hemical requirements for the RNA photocleavage were investigated. As shown in Fig. 2A, the DNA strand worked efficiently even without magnesium ion in presence of 200 μM riboflavin (lane 1), and the cleavage efficiency did not change too much with different concentrations of Mg^2+^ (Lane 2–4). However, the catalytic activity of the DNA strand strongly depended on the concentration of riboflavin (Fig. 2B). The photocleavge of RNA target by the DNA strand increased along with the increase of riboflavin concentration, reaching the maximal activity in around 50 μM (Fig. 2B). Moreover, the temperature has little influence to the cleavage efficiency based on the DNA strand in combination with riboflavin from 20 to 40 ^o^C (data no shown). Under optimal condition, the DNA strand in combination with riboflavin catalyzed the photocleavage of target RNA with a *k*_obs_ as high as 0.25 h^−1^, the half life being around 6 hours, which is much faster than most of reported artificial ribonucleases^2^. The RNA substrate containing **XUY** for this strategy has been carefully studied (Supplementary Fig. S1). The nucleoside **X** located at the 5′ of the uracil of the G•U base pair could affect the cleavage efficiency of RNA substrates (G ≈ A > C > U). The nucleoside **Y** RNA in substrate that will be removed from 3′ of U revealed the tread of C > U > A > G in terms of cleavage efficiency (Supplementary Fig. S1). The RNA substrate for the sequence-specific photocleavage has the frequency of one repeat in every 4 bp, which reveals the broad generality of this molecular tool with respect to different RNA sequence. The length of two binding arms of the DNA strand is variable and the catalytic efficiency would not be affected as long as the total length of two binding arms are longer than 12 nt (Supplementary Fig. S2).

As comparison, the RNA hairpin (35 nt in length) that encodes the same sequence of RNA target at 5′end was applied to self-cleavage under the same conditions. Both the intramolecular and intermolecular reaction provided the cleaved products with the same length (Lane 2 and Lane 4, Fig. 3C), revealing riboflavin could bind to the G•U wobble base pair in RNA/RNA as well as RNA/DNA duplexes and photocleave the RNA target sequence with similar efficiency. However, the cleaved product has slightly faster mobility than the 5-^32^P-labeled RNA marker (17 nt), revealing the presence of one more phosphate group at the 3′-end (Lane 1 and Lane 2, Fig. 3C). Therefore, T4 polynucleotide kinase (PNK) that could remove phosphate group from 3′-end of RNA has been added to the reaction mixture. The treatment with PNK didn’t change the mobility of cleaved fragment on gel (Lane 2 and Lane 4, Fig. 3C), indicating that the cleavage reaction could go through oxidative elimination mechanism to form unnatural 3′-phosphate RNA terminal. Moreover, the photocleavage activity reduce 74% in the absence of oxygen (Fig. 3D), revealing the excited photosensitizer interacts with ground state oxygen to generate a singlet oxygen molecule, which is the same as what Famlok group has observed in the photocleavage of RNA duplex^19^.

To validate the scope of this method, mircoRNA21 with 22 nt in length was applied as the target to verify whether our photocleavage method based on the DNA strand in combination with riboflavin can work or not. An uracil (U) in the middle of mircoRNA21 was selected as the cleaving site, and the DNA strand was designed to complement to the target sequence except for the formation of a G•U wobble (Fig. 4A). As shown in the isotope-labeled experiment, a cleaved fragment with expected length (11 nt) was detected based on the PAGE analysis of the photo-induced cleavage reaction (Lane 3 Fig. 4B). For the longer RNA target, we can design multiplex cleaving-sites to improve the cleavage efficiency and specificity based on our method. As a proof-of-concept, three uracil (U) of RNA (Long-RNA, Fig. 4C) has been targeted by DNA strand, and any of them once cleaved by DNA strands could result in the disfunction of RNA target. As comparison, RNA (Short-RNA, Fig. 4C) with only one cleaving-site was cleaved by one DNA strand under same reaction condition. After 2 h of photocleavage in presence of riboflavin, more than 53% of the longer RNA containing three cleaving-sites was cleaved, while only 28% of cleavage obtained for the reaction of the shorter RNA (Lane 3 and Lane 6, Fig. 4D).

To further confirm the DNA stand in combination with riboflavin as tool to hinder the RNA function through cleavage, RNA mimic of GFP (Green Fluorescent Protein) was chosen as the object of study. Recently, Jaffrey group has described a complex of RNA aptamer (24–2) and a derivative of the GFP fluorophore, 3,5-difluoro-4-hydroxybenzylidene imidazolinone (DFHBI), termed as “Spinach” that emits fluorescence upon transformation in specific conformation^21^ (Fig. 5A). This RNA-fluorophore complex displays fluorescence properties similar to GFP and is unlike most fluorescent proteins, resistant to photo-bleaching and conditionally fluorescent. We designed DNA strand (24–2-Cat) to cleave RNA 24–2 through forming G•U wobble base pair at 31 nt position of the uracil (U) (Fig. 5B), and the cleavage activity of 24–2-Cat to RNA target in presence of riboflavin was verify by the following isotop-labeled experiment and PAGE analysis (Lane 3 Fig. 5C). The fluorescence intensity of the complexes (24–2 + DFHBI) was significant (blue curve, Fig. 5D). However, by adding 24–2-Cat into the reaction, the fluorescence intensity dropped to 44.61% after 5 h photocleavage in comparison with the reaction without the DNA strand (green curve, Fig. 5D). As a control, full complementary DNA sequence (24–2-NC, Fig. 5B) was introduced into the same reaction as a comparison, showing only 7.59% fluorescence intensity decrease (red curve, Fig. 5D). Those results indicates the photocleavage can directly hinder the function of RNA target through cleavage other than block the RNA target by forming DNA/RNA duplex.

## Discussion

The established sequence-specific photocleavge of RNA described here appears to have several promising features to become a new molecular tool for research and application as follows: (1) the designation of DNA strand to realize the seqence specific cleavage of RNA target is very easy to fulfill only if there are uracil nucleotides in the RNA sequence and the DNA strand is complementary to the target sequence expect to form a G•U wobble in between. (2) Though the minimal size of DNA strand is 13 nt in length, high sequence specificity could be achieved by increase the length of DNA stand to form the longer DNA/RNA duplex with target. The efficiency of the cleavage reaction based on the DNA strand in combination with riboflavin is very high. And the cleaving specificity and efficiency could be further improved by introducing more strands to cleave the same RNA at multiplex cites. (3) The cleavage of RNA by DNA strand is under tight control by using light as switch, which means the cleavage could be carried out according to specific spatial and temporal requirements. Moreover, the photosensitizer, riboflavin (vitamin B2), is endogenic coenzyme existing in the cell of most living creature, which makes this method possible to apply *in vivo*. (4) Unlike antisense oligonucleotide, the DNA strand in our method could interfere with the function of RNA through the irreversible photocleavge, forming cleaved fragments with unnatural 3′ -phosphate RNA terminal.

In summary, we developed a new RNA-cleaving DNA strand that catalyzes the sequence-specific photo-induced cleavage of RNA at the 3′ of the uracil of G•U wobble base pair in the presence of rioflavin as cofactors. Unlike most artificial ribonuclease that need conjugate functional chemical group to DNA sequence to fulfill the cleavage of RNA molecules specifically, the DNA strand described here is unmodified DNA sequence, cooperating with riboflavin to realize the photocleavage of RNA target through oxidative elimination mechanism. This method has broad substrate scope if the targeted RNA contains required uracil nucleotide that has high frequency of one repeat in every 4 bp. The function of RNA molecules were successfully hinder by photocleavage based on our method *in vitro*, which was proved to proceed through the cleavage reaction other than the hybridization with target. The biocompatibility of this method makes the DNA strand in combination with riboflavin a promising manipulable molecular tool *in vivo*. The direct conjunction the DNA strand with riboflavin and the application our method in the cell experiment is underway in our lab.

## Additional Information

**How to cite this article**: Zhao, Y. *et al.* Sequence-specific RNA Photocleavage by Single-stranded DNA in Presence of Riboflavin. *Sci. Rep.* **5**, 15039; doi: 10.1038/srep15039 (2015).

## Supplementary Material

Supplementary Information

## Figures and Tables

**Figure 1:**
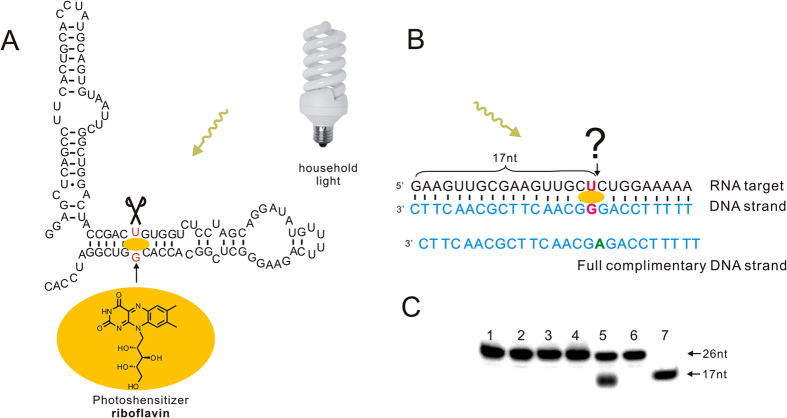
(**A**) Secondary structure of the RNA aptamer obtained through *in vitro* selection by Famulok group. One cleavage sites at the G•U wobble pair present in this RNA is marked with red color. (**B**) RNA/DNA duplex containing one G•U wobble pair. (**C**) PAGE analysis of the cleavage of 5′ ^32^P-RNA target by the DNA strand in 250 mM NaCl, 50 mM Tris-HCl, pH 7.6, 12 mM MgCl_2_ and 200 μM riboflavin under irradiation with 45 W h household light for 4 h at 25 ^o^C. Lane 1: Cleaving reaction without the DNA strand; Lane 2: Cleaving reaction with riboflavin; Lane 3: Cleaving reaction without light irradiation; Lane 4: cleavage of 5′ ^32^P-labeled RNA by using full complementary DNA; Lane 5: cleaving reaction by the DNA strand; Lane 6: 5′ ^32^P-labeled RNA target (26 nt); Lane 7: 5′ ^32^P-labeled RNA marker (17 nt). Chemical structure of riboflavin used for photoinduced RNA cleavage is illustrated in the orange region.

**Figure 2:**
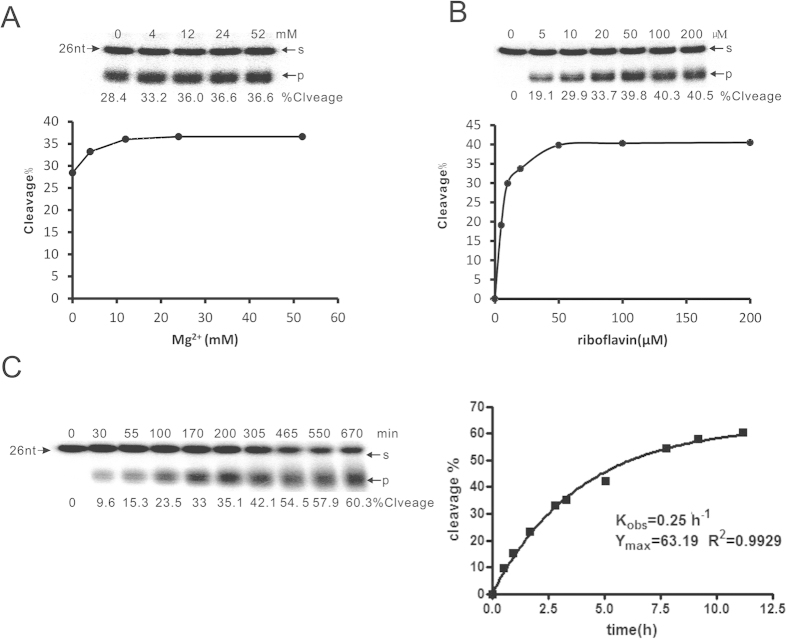
The analysis of RNA photocleavage reactions under different conditions. (**A**) Catalytic rates were determined in the presence of saturating substrate (100 nM) with varying concentrations of MgCl_2_ (0–52 mM). Reaction conditions were otherwise as above. (**B**) Catalytic rates were determined in the presence of saturating substrate (100 nM) with varying concentrations of riboflavin (0–200 μM). (**C**)Kinetic plots of DNA with GraphPad Prism 4 and PAGE analysis of cleaving reactions catalyzed by DNA. S: un-cleaved RNA substrates; P: cleaved fragment.

**Figure 3:**
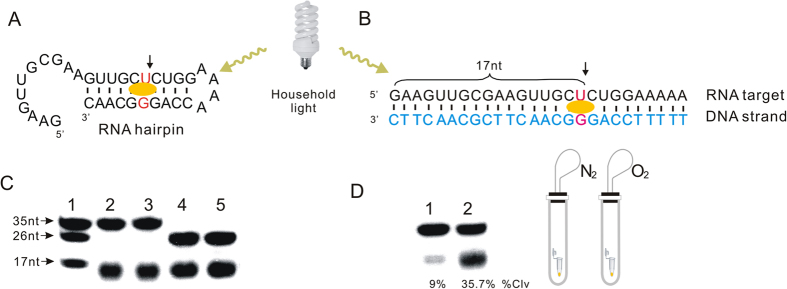
Mechanism study of RNA photocleavage. (**A**) RNA haipin (35 nt) containing a G•U wobble pair which is marked in red. (**B**) RNA/DNA duplex formed between RNA target (26 nt) and the DNA strand. (**C**) PAGE analysis of cleavage reactions. Lane 1: 5′ ^32^P-labeled RNA markers (35 nt, 26 nt, 17 nt); Lane 2: Intramolecular cleavage reaction of RNA haipin; Lane 3: Treatment of the intramolecular cleavage reaction with PNK; Lane 4: Intermolecular cleavage of RNA target with the DNA strand; Lane 5 Treatment of the intermolecular cleavage reaction with PNK. (**D**) PAGE analysis of cleavage reaction under protection with nitrogen (N_2_) or oxygen (O_2_) environment. Lane 1: cleavage reaction in the present of N_2;_ Lane 2: cleavage reaction in the present of O_2._

**Figure 4:**
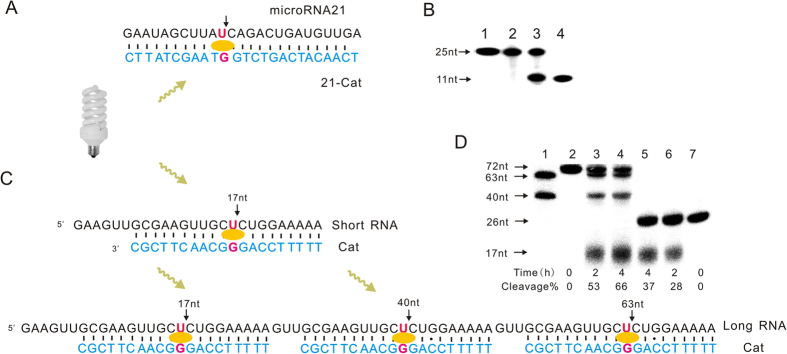
The photocleavge different RNA targets. (**A**) microRNA21 was cleaved with the DNA strand 21-Cat. (**B**) PAGE analysis of cleavage of 5′ ^32^P-labeled microRNA21 (25 nt). Lane 1: 5′ ^32^P-labeled RNA marker (25 nt); Lane 2: Cleaving reaction without 21-Cat; Lane 3: Cleavage microRNA21 by using 21-Cat; Lane 4: 5′ ^32^P-labeled RNA marker (11 nt). (**C**) RNA targets contain one (Short-RNA) and three (Long-RNA) cleaving cites were photocleaved. (**C**) PAGE analysis of cleavage of 5′ ^32^P-labeled Short-RNA (26 nt) and Long-RNA (72 nt). Lane 1: 5′ ^32^P-labeled markers (63 nt, 40 nt); Lane 2–4: The cleaving reaction of 5′ ^32^P-labeled Short-RNA for 0 h, 2 h and 4 h in presence of the DNA strand Cat; Lane 5–7: The cleaving reaction of 5′ ^32^P-labeled Long-RNA for 0 h, 2 h and 4 h in presence of the DNA strand Cat.

**Figure 5:**
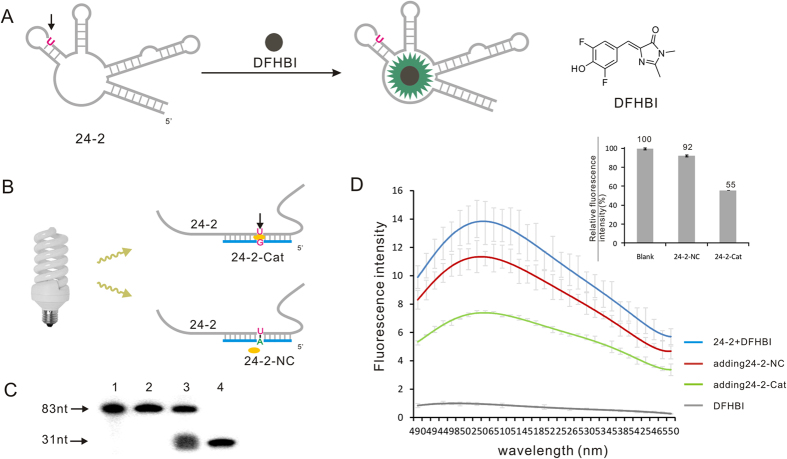
Hindering RNA function by used the photocleavage strategy. (**A**) The complex of RNA aptamer (24–2) and fluorophore DFHBI reveals similar fluorescent properties as GFP. (**B**) The DNA strand 24–2-Cat was designed to cleave RNA 24–2 through forming G•U wobble base pair at 31 nt position; The DNA strand 24–2-NC was full complementary to the same domain of RNA 24–2. (**C**) PAGE analysis of cleavage reaction. Lane 1: 5′ ^32^P-labeled 24–2 (83 nt); Lane 2: cleaving 24–2 by using 24–2-NC; Lane 3: Cleaving 24–2 with 24–2-Cat; Lane 4: 5′ ^32^P-labeled RNA marker (31 nt). (**D**) Result of the treatment with 24–2-Cat on RNA-fluorophore complexes in *in-vitro*. The fluorescnet intensity was recorded after 5 h photocleavge reaction, and the insert shown the relative quantitative fluorescent intensity.

## References

[b1] Trawick B. N., Daniher A. T. & Bashkin J. K. Inorganic Mimics of Ribonucleases and Ribozymes: From Random Cleavage to Sequence-Specific Chemistry to Catalytic Antisense Drugs. Chem Rev 98, 939–960 (1998). 11848920 10.1021/cr960422k

[b2] Niittymaki T. & Lonnberg H. Artificial ribonucleases. Organic & biomolecular chemistry 4, 15–25 (2006). 16357988 10.1039/b509022a

[b3] Murtola M., Wenska M. & Stromberg R. PNAzymes that are artificial RNA restriction enzymes. Journal of the American Chemical Society 132, 8984–8990 (2010). 10.1021/ja1008739 20545354

[b4] Nakano S., Uotani Y., Uenishi K., Fujii M. & Sugimoto N. Site-selective RNA cleavage by DNA bearing a base pair-mimic nucleoside. Journal of the American Chemical Society 127, 518–519 (2005). 10.1021/ja045445s 15643864

[b5] Hall J., Husken D. & Haner R. Towards artificial ribonucleases: the sequence-specific cleavage of RNA in a duplex. Nucleic acids research 24, 3522–3526 (1996). 8836177 10.1093/nar/24.18.3522 PMC146125

[b6] Wu H., Hait W. N. & Yang J. M. Small interfering RNA-induced suppression of MDR1 (P-glycoprotein) restores sensitivity to multidrug-resistant cancer cells. Cancer Res. 63, 1515–1519 (2003). 12670898

[b7] Martinez J., Patkaniowska A., Urlaub H., Luhrmann R. & Tuschl T. Single-stranded antisense siRNAs guide target RNA cleavage in RNAi. Cell 110, 563–574 (2002). 10.1016/s0092-8674(02)00908-x 12230974

[b8] Meister G. & Tuschl T. Mechanisms of gene silencing by double-stranded RNA. Nature 431, 343–349 (2004). 15372041 10.1038/nature02873

[b9] Scott W. G., Murray J. B., Arnold J. R. P., Stoddard B. L. & Klug A. Capturing the structure of a catalytic RNA intermediate: The hammerhead ribozyme. Science 274, 2065–2069 (1996). 8953035 10.1126/science.274.5295.2065

[b10] Birikh K. R., Heaton P. A. & Eckstein F. The structure, function and application of the hammerhead ribozyme. European Journal of Biochemistry 245, 1–16 (1997). 10.1111/j.1432-1033.1997.t01-3-00001.x 9128718

[b11] Dass C. R., Saravolac E. G., Li Y. & Sun L. Q. Cellular uptake, distribution, and stability of 10-23 deoxyribozymes. Antisense & Nucleic Acid Drug Development 12, 289–299 (2002). 12477279 10.1089/108729002761381276

[b12] Silverman S. K. *In vitro* selection, characterization, and application of deoxyribozymes that cleave RNA. Nucleic Acids Research 33, 6151–6163 (2005). 16286368 10.1093/nar/gki930 PMC1283523

[b13] Grate D. & Wilson C. Laser-mediated, site-specific inactivation of RNA transcripts. Proc. Natl. Acad. Sci. USA. 96, 6131–6136 (1999). 10.1073/pnas.96.11.6131 PMC26847 10339553

[b14] Kasai H., Yamaizumi Z., Berger M. & Cadet J. Photosensitized Formation Of 7,8-Dihydro-8-Oxo-2′-Deoxyguanosine (8-Hydroxy-2′-Deoxyguanosine) In Dna By Riboflavin - A Nonsinglet Oxygen Mediated Reaction. J. Am. Chem. Soc. 114, 9692–9694 (1992).

[b15] Kino K., Saito I. & Sugiyama H. Product analysis of GG-specific photooxidation of DNA via electron transfer: 2-aminoimidazolone as a major guanine oxidation product. J. Am. Chem. Soc. 120, 7373–7374 (1998).

[b16] Ito K., Inoue S., Yamamoto K. & Kawanishi S. 8-hydroxydeoxyguanosine formation at the 5′ site of 5′-gg-3′ sequences in double-stranded dna by uv-radiation with riboflavin. J. Biol. Chem. 268, 13221–13227 (1993). 8390459

[b17] Frier C., Mouscadet J. F., Decout J. L., Auclair C. & Fontecave M. Flavin-oligonucleotide conjugates: sequence specific photocleavage of DNA. Chem. Commun., 2457–2458 (1998).

[b18] Burgstaller P. & Famulok M. Isolation Of Rna Aptamers For Biological Cofactors By *In-Vitro* Selection. Angew. Chem.-Int. Edit. Engl. 33, 1084–1087 (1994).

[b19] Burgstaller P. & Famulok M. Flavin-dependent photocleavage of RNA at G center dot U base pairs. Journal of the American Chemical Society 119, 1137–1138 (1997).

[b20] Burgstaller P., Hermann T., Huber C., Westhof E. & Famulok M. Isoalloxazine derivatives promote photocleavage of natural RNAs at G center dot U base pairs embedded within helices. Nucleic Acids Research 25, 4018–4027 (1997). 9321652 10.1093/nar/25.20.4018 PMC146990

[b21] Paige J. S., Wu K. Y. & Jaffrey S. R. RNA Mimics of Green Fluorescent Protein. *Science* 333, 642–646 (2011). 10.1126/science.1207339 PMC3314379 21798953

