# Supplementary material for: Sequence-specific RNA Photocleavage by Single-stranded DNA in Presence of Riboflavin
Source: Sci Rep. 2015 Oct 13;5:15039. doi: 10.1038/srep15039 (PMC4602204; doi:10.1038/srep15039)
Supplement: Supplementary Information [file srep15039-s1.pdf]

Supplementary Information

General information. T7 RNA polymerase, FastAP Thermosensitive Alkaline Phosphatase (FastAP), T4 PNK were purchased from MBI Fermentas. NTP were purchased from TransGen Biotech (Beijing, China). [a-^{32}P]dATP was purchased from Furui Biological Engineering (Beijing, China). Riboflavin was purchased from Sangon Biotech (Shanghai, China); DFHBI was synthesized by ourself and all oligonucleotides were purchased from Sangon Biotech (Shanghai, China) and purified by PAGE.

RNA transcription. RNA substrates were synthesized by RNA transcription using T7 RNA polymerase and a double-stranded DNA template having the following sense sequence 5-GAATT *CTAATACGACTCACTATA*-RNA-3 (italicized letters indicates the promoter sequence for T7 RNA polymerase). A transcription reaction containing 0.5 mM of DNA template, 10 mM of NTP, T7 RNA polymerase buffer (40 mM Tris–HCl (pH 7.9), 10 mM NaCl, 10 mM dithiothreitol, 6 mM $MgCl_2$, 2 mM spermidine) and 1.25 unit per ml RNA polymerase were incubated at 25° C for 4 h, followed by ethanol precipitation and purification by PAGE.

Labelling reaction. A reaction mixture containing oligonucleotides with 50 mM Tris–HCl (pH 7.8), 40 mM NaCl, 10 mM $MgCl_2$, 1 mg/ml BSA, 10 mCi[a-^{32}P]ATP and 10 units of PNK was incubated for 1 h at 37° C. The labelled product was purified by 10% denaturing PAGE. Single stranded DNA could be labeled according to this method directly, but RNA substrates obtained by in-vitro transcription must be treated with alkaline phosphatase before the labeling experiment as follows: RNA was incubated with 0.05 unit per ml FastAP in 10 mM Tris–HCl (pH 8.0), 5 mM $MgCl_2$, 100 mM KCl, 0.02% Triton X-100 and 0.1 mg/ml BSA at 37° C for 10 min, and then extracted with phenol-chloroform and recovered by ethanol precipitation.

Kinetic analysis of DNA strand. Cleavage reaction containing 1 μM DNA, 150 nM 5-^{32}P-labelled RNA, 200 mM NaCl, 50 mM Tris–HCl, pH 7.6, 2 mM EDTA, 12 mM $MgCl_2$ and 200 μM riboflavin was incubated at 25° C using incident light from a polychromatic lamp (40W) at a distance about 15 cm from the sample. The reaction was stopped after a designated period of time, by adding EDTA (pH 8.0) to a final concentration of 30 mM. Cleavage products were separated by 10% denaturing PAGE and quantified by phosphorimager (PerkinElmer Cyclone Plus Storage PhosphorSystem). Time courses for each deoxyribozyme were conducted at least twice, using over eight time points for each. The experimental data was fit to the $Y=Y_{max}(1-e^{(k\ obst)})$ exponential equation using non-linear regression analysis in GraphPad Prism 4, from which the observed rate constant (K_{obs}) and maximum cleavage yield (Y_{max}) were determined.

Photocleavage experiment. A cleavage reaction mixture containing 1 μM DNA, 150 nM 5-^{32}P-labelled RNA, 200 mM NaCl, 50 mM Tris–HCl, pH 7.6, 2 mM EDTA, 12 mM $MgCl_2$ and 200 μM riboflavin was incubated at 25° C. for 5 h using incident light from a polychromatic lamp (40W) at a distance about 15 cm from the sample. All reactions were analyzed by using the 20% denaturing PAGE.

The cleavage the RNA mimic of GFP. A cleavage reaction mixture containing 1 μM DNA, 0.5 μM 24-2, 200 mM NaCl, 50 mM Tris–HCl, pH 7.6, 2 mM EDTA, 12 mM $MgCl_2$ and 150 μM riboflavin was incubated at 25° C for 5h using incident light from a polychromatic lamp (40W) at a distance about 15 cm from the sample and then recovered by ethanol precipitation. Add 200 mM NaCl, 50 mM Tris–HCl, pH 7.6, 2 mM EDTA, 12 mM $MgCl_2$ and 200 μM DFHBI. Fluorescence emission measurements with DFHBI as the fluorophore were carried out on a Cary Eclipse Device (excitation 469 nm, emission 501 nm; Varian Inc).

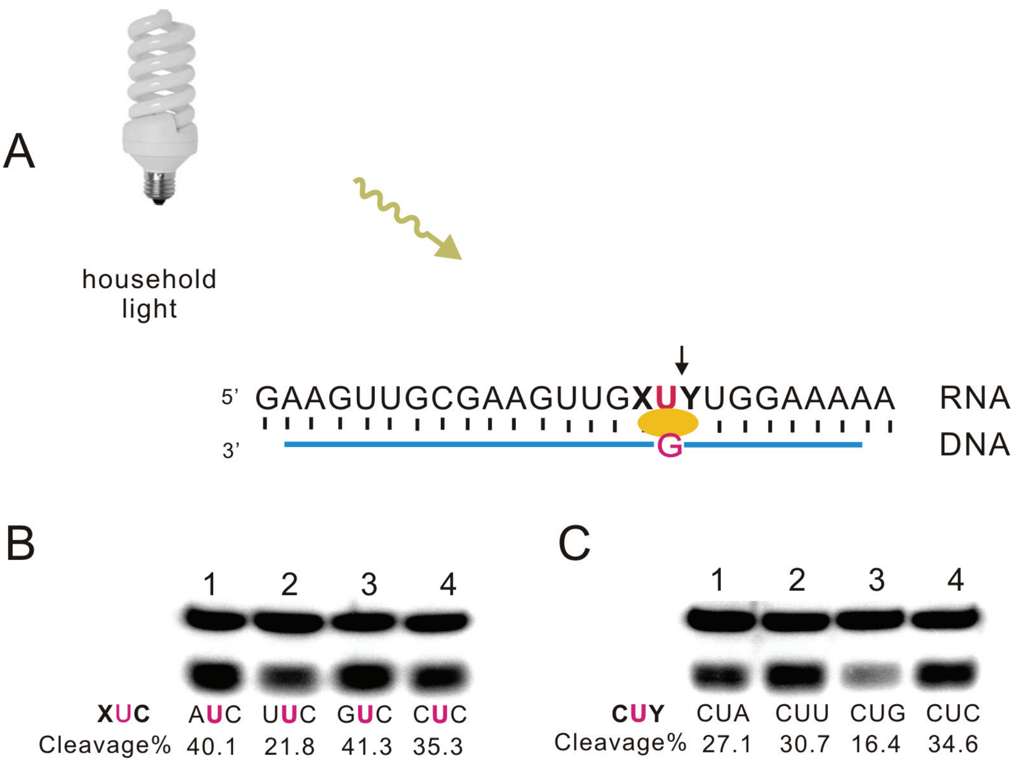

Figure S1: **The investigation of the cleavage efficiency of different RNA substrate on photocleavage** by DNA strand in presence of riboflavin (A) The RNA Sequences containing **XUY** were cleaved by corresponding DNA strand based on our method. (B) The cleavage efficiency of RNA substrates containing the **X**UC was studied based on the PAGE analysis of isotope-labeled experiment. (C) The cleavage efficiency of RNA substrates containing the CU**Y** was studied based on the PAGE analysis of isotope-labeled experiment.

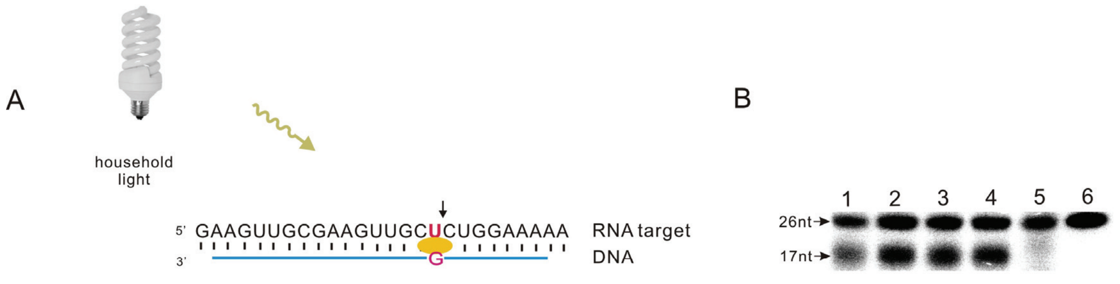

C

PAGE	1	2	3	4	5
Arm length	16+9	9+9	8+8	7+7	6+6
Cleavage yield	49.6%	45.5%	46.6%	47.3%	---

Figure S2. PAGE analysis of the effect of the length binding arms on the cleavage effiency. (A) RNA taget would be cleaved by different DNA strands with varying binding arms. (B) PAGE analysis of cleavage of RNA substrate based on isotope-labeled experiments. Lane1-Lane5: cleavage of ^{32}P-labelled RNA by DNA with varying different length of binding arms; Lane 6: marker, 5-^{32}P-labelled RNA substrate (26 nt). (C) The stable revealing the cleaving yields by DNA strands with different binding arms.

Supplimentary Table S1: Sequence information

Identity	Sequences
**RNA hairpin**	GAAGUUGCGAAGUUGCUCUGGAAAACCAGGGCA AC
**Short RNA**	GAAGUUGCGAAGUUGCUCUGGAA AAA
**Long RNA**	GAA GUU GCGAA GUU GCU CUG GAA AAA GUU GCGAA GUU GCU CUG GAA AAA GUU GCGAA GUU GCU CUG GAA AAA
**Cat**	TTT TTCCAGGGCAACTTCGC
**microRNA21**	GAAUAGCUUAUCAGACUGAUGUUGA
**21-Cat**	TCA ACATCAGTCTGGTAAGCTATTC
**24-2**	GGGAGACGCAACUGAAUGAACCUAGAGUUAUGCCAGGCUCUGAGCCUGCUUCGGCAGGUGCUAUGAUCGCCAGCGGUAUGCAGUCCGUAACUAGUCGCGUCAC
**24-2-NC**	GAAGCACTGGACCCGTCCTTC
**24-2-Cat**	GAAGCACTGGGCCCGTCCTTC
